# Effects of Anisotropy and In-Plane Grain Boundary in Cu/Pd Multilayered Films with Cube-on-Cube and Twinned Interface

**DOI:** 10.1186/s11671-021-03528-9

**Published:** 2021-04-28

**Authors:** Xiang Chen, Shayuan Weng, Xing Yue, Tao Fu, Xianghe Peng

**Affiliations:** 1 College of Mechanical Engineering, Chongqing University, Chongqing, 400044 China; 2 Department of Engineering Mechanics, Chongqing University, Chongqing, 400044 China; 3 State Key Laboratory of Coal Mine Disaster Dynamics and Control, Chongqing University, Chongqing, 400044 China

**Keywords:** Interfacial structure, Anisotropy of mechanical properties, Strengthening effect, In-plane polycrystalline

## Abstract

In crystalline materials, grain boundary and anisotropy of crystal structure affect their mechanical properties. The effects of interfacial structure on the mechanical properties may be diverse when the multilayer film is loaded along different directions. In this work, we performed a series of molecular dynamics simulations of the tension of in-plane single and polycrystalline Cu/Pd multilayered films with cube-on-cube (COC) and twinned interfaces to explore the effects of the interfacial structure, loading direction and in-plane grain boundaries on their mechanical properties. The interfacial misfit dislocation lines become bent after relaxation, and the high temperature of 300 K was found as a necessary condition. When stretched along 〈110〉 direction, the strengthening effect of the COC interface is more noticeable; however, when stretched along 〈112〉 direction, the twin interface's strengthening effect is more visible, showing the anisotropic effect of interfacial structure on mechanical properties. However, in the in-plane honeycomb polycrystalline sample, the twin interface showed a pronounced strengthening effect, and no jogged dislocations were observed.

## Introduction

Nanostructured metallic multilayered (NMM) films have attracted much attention due to their excellent mechanical properties [1–3], which are usually superior to their constituents. The interface, transition zone between different individual layers, is one of the most common planar defects in NMM films, which can act as sources and sinks of defects via absorption and annihilation, barriers and storage sites for defects [4–7].

The interfaces in an NMM film can be divided into a coherent, semi-coherent and non-coherent interface based on the lattice mismatch between their constituents on both sides of the interface [4]. Copper-palladium (Cu/Pd) and gold–nickel (Au/Ni) multilayered films are the earliest found multilayered films possessing excellent mechanical properties[8]. Yang et al. measured the biaxial elastic modulus Y[111] of Cu/Pd and Au/Ni multilayered films by bulge testing and found their biaxial elastic modulus increases drastically from 0.27 to 1.31 TPa and from 0.21 to 0.46 TPa, respectively [8]. Subsequently, Davis et al*.* used more advanced techniques to measure elastic and structural properties of Cu/Pd and Cu/Ni multilayered films with the same growth textures and composition modulation amplitudes [9, 10]. However, no significant anomalous elastic behavior has been observed [9, 10], which raises whether the supermodulus effect exists in the Cu/Pd multilayers. The mechanical properties of NMM are strongly dependent on the interfacial structure between adjacent individual layers [11]. Howe et al. investigated the interfacial structure of Pd films on Cu(111) and found that the Pd grows in a twinned FCC structure along 〈111〉 direction [12]. The twinning structures at the interface usually have a profound effect on their strength [11].

Weng et al*.* investigated the effect of interfacial structure on the deformation behaviors of Cu/Ni multilayered films with coherent, semi-coherent and coherent twin interfaces using molecular dynamics (MD) simulation and found that the coherent twin interface shows significant strengthening [7]. However, in our recent work, the inapparent strengthening effect of the twin interface in Cu/Pd multilayered films was observed under tension along 〈110〉 direction [13]. Besides, the shape of the misfit dislocation network would change during energy minimization and relaxation. Shao et al. investigated the relaxation mechanisms of interfaces and the evolution of interfacial dislocation networks in the Cu/Ni multilayered films by MD simulations [14–17]. These works' loading direction is often perpendicular to the interface, referred to as out-of-plane [7, 18, 19]. However, the interface may play different roles during loading along different directions due to the anisotropy of the mechanical properties of crystals [20–23].

Besides, multilayered films are more inclined to be subjected to the load parallel to the interface in practice, referred to as in-plane loading. Zhou et al. proposed a strengthening mechanism governed by multiple necklace-like extended jogged dislocations in a columnar-grained nano-twinned metal subjected to external stress paralleled to the twin planes [20], which is also observed in Cu/Ni multilayer [21]. These jogged dislocations are rarely found in a simulation under an out-of-plane loading [7, 18, 19, 24]. In available MD simulations of in-plane tensions, the sample is usually stretched along a specific direction, i.e., 〈112〉 or 〈110〉 direction [25]. However, few comparative studies under tension along these two directions have been conducted. On the other hand, the individual layer of the multilayered film prepared by experiments is usually in-plane polycrystalline containing many grain boundaries (GBs) perpendicular to the interface.

The jogged dislocations mentioned above are often observed in the coherent twined films or twined multilayered films with a minor mismatch. Whether these jog dislocations can form in a twin interface film with a high mismatch is still unknown. The Cu/Pd multilayered film is the earliest found multilayered film having excellent mechanical properties [8, 12, 26–28]. Its lattice mismatch (~ 7.07%) is larger than that of Cu/Ni multilayered films (~ 2.7%). Therefore, the strengthening and weakening mechanism [7, 14–17] obtained by the Cu/Ni multilayered film may not be applied to the Cu/Pd multilayered film. Two common interfaces [3], twin and cube-on-cube interface, are observed in Cu/Pd multilayered film by experimental characterization [12]. Understanding the effect of interfacial structure on multilayered films' mechanical properties would be significant for designing high-performance nano-multilayer films with a large lattice mismatch.

In this work, two types of samples with in-plane honeycomb crystal and single-crystal are developed. For each type of sample, two kinds of interfaces (cube-on-cube and twin) are considered. Then we perform a series of MD tension simulations of these Cu/Pd multilayered films to explore the effects of the interfacial structure, loading direction, and in-plane GBs on their mechanical properties.

## Methods

Three sets of parameters for Cu–Cu, Pd–Pd, and Cu–Pd are needed to be identified, respectively. We choose the second nearest-neighbor modified embedded atom method (2NN MEAM) potential [29, 30] to describe the interactions between atoms. For the Cu–Cu and Pd–Pd, their potential parameters have been developed by Lee et al. [31]. Based on the single elements' potential parameters, we fitted a set of Cu–Pd binary potential parameters in our previous work [26], as listed in Table 1. These parameters can reproduce the fundamental physical and mechanical properties of pure Cu, Pd and their alloys and describe the formation mechanism of growth twins [26].

**Table 1:** 2NN MEAM potential parameters for the Cu-Pd system [26]. *E*_c_, *r*_e_, and B are cohesive energy, equilibrium nearest-neighbor distance and bulk modulus of B2 CuPd alloy

Parameter	*E*_*c*_ (eV)	*r*_*e*_ (Å)	_*B*_ (GPA)	_*d*_	Cu–Pd–Cu		Pd–Cu–Pd		Cu–Cu–Pd		Pd–Pd–Cu	
					*C*_min_	*C*_max_	*C*_min_	*C*_max_	*C*_min_	*C*_max_	*C*_min_	*C*_max_
Value	3.725	2.593	106.2	0.05	0.65	1.44	0.78	1.44	1.44	2.8	1.44	2.8

**Fig. 1:**
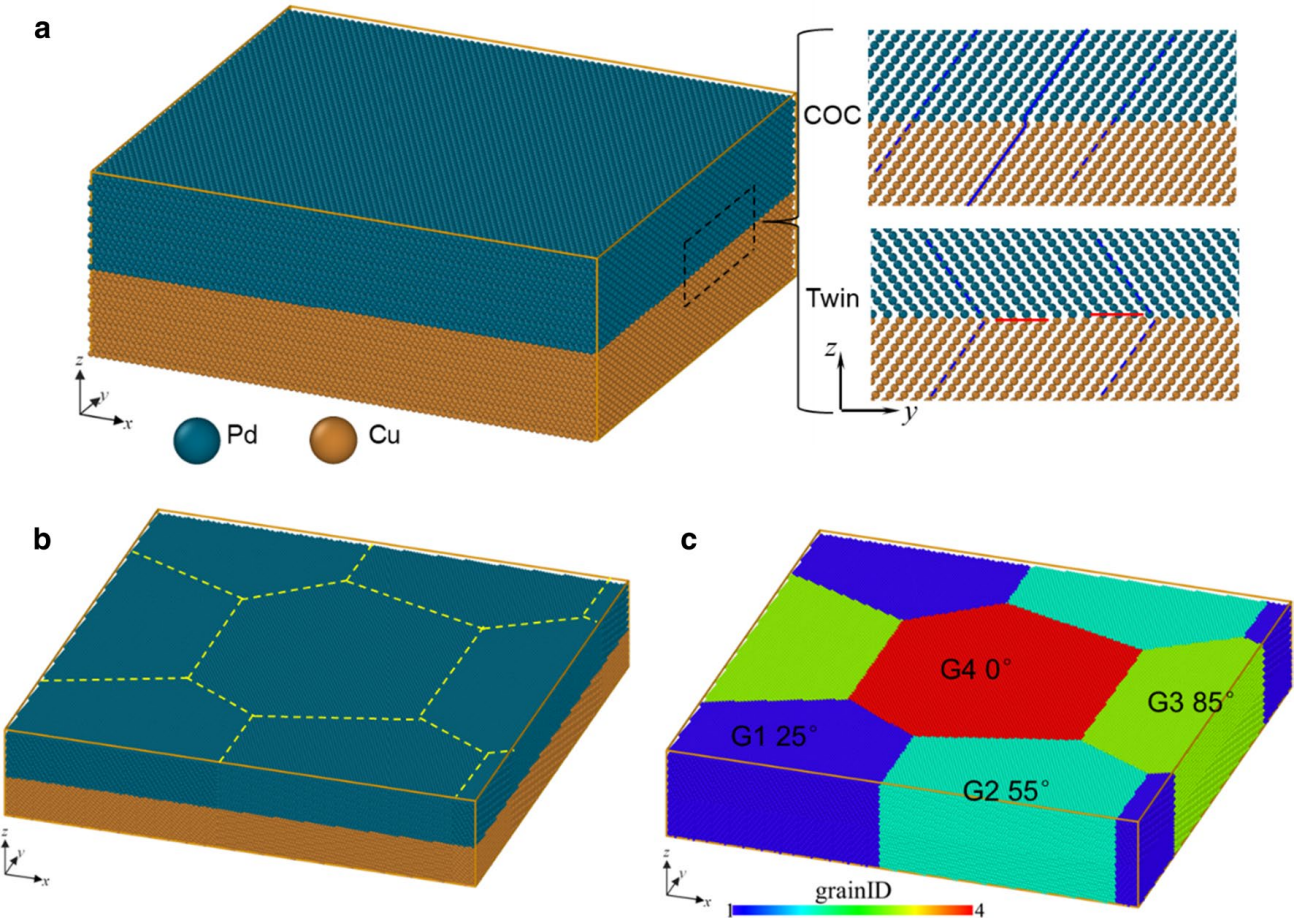
The atomic model with in-plane **a** single crystal and **b** honeycomb crystal. **c** The orientation relationships of each grain concerning the single crystal. The insets of Fig. 1a are the atomic distribution of COC and Twin interfaces, where the red lines represent twins

The FCC/FCC multilayered film is prone to grow along 〈111〉 directions and the orientation relationship of the interface is identified as {111}_FCC_/{111}_FCC_ [32, 33]. Therefore, we only consider the Cu{111}/Pd{111} interfaces in this work. Two types of samples with in-plane single crystal (SC) and honeycomb crystal (HC) are built, as shown in Fig. 1a and b. For each type of sample, cube-on-cube (COC) and twin interface are considered. Therefore, four samples are built, named SC COC, SC Twin, HC COC and HC Twin. For SC COC, the crystal orientations of the Cu layer and the Pd layer are identical; however, for SC Twin, their crystal orientations are symmetric about the twin interface, as shown in the inset of Fig. 1a. The orientation relations and dimensions of each direction are listed in Table 2.

**Table 2:** Crystal orientations and sizes of each direction of samples. *a*_Cu_ and *a*_Pd_ are the lattice parameters of Cu and Pd (3.615 Å and 3.890 Å)

In-plane	Sample	Constituent	*l*_*x*_	*l*_*y*_	*l*_*z*_	Model
Single-crystal (SC)	COC	Pd layer	$80 \times \frac{1}{2}[01\overline{1}]a_{{{\text{Pd}}}}$	$40 \times \frac{1}{2}[\overline{2}11]a_{{{\text{Pd}}}}$	$18 \times \frac{1}{3}[111]a_{{{\text{Pd}}}}$	_SC COC_
		Cu layer	$86 \times \frac{1}{2}[01\overline{1}]a_{{{\text{Cu}}}}$	$43 \times \frac{1}{2}[\overline{2}11]a_{{{\text{Cu}}}}$	$18 \times \frac{1}{3}[111]a_{{{\text{Cu}}}}$	
	Twin	Pd layer	$80 \times \frac{1}{2}[01\overline{1}]a_{{{\text{Pd}}}}$	$40 \times \frac{1}{2}[\overline{2}11]a_{{{\text{Pd}}}}$	$18 \times \frac{1}{3}[\overline{1}\overline{1}\overline{1}]a_{{{\text{Pd}}}}$	_SC Twin_
		Cu layer	$86 \times \frac{1}{2}[01\overline{1}]a_{{{\text{Cu}}}}$	$43 \times \frac{1}{2}[\overline{2}11]a_{{{\text{Cu}}}}$	$18 \times \frac{1}{3}[111]a_{{{\text{Cu}}}}$	
Honeycomb crystal (HC)	COC	Pd layer	400 Å	400 Å	$18 \times \frac{1}{3}[111]a_{{{\text{Pd}}}}$	_HC COC_
		Cu layer	400 Å	400 Å	$18 \times \frac{1}{3}[111]a_{{{\text{Cu}}}}$	
	Twin	Pd layer	400 Å	400 Å	$18 \times \frac{1}{3}[\overline{1}\overline{1}\overline{1}]a_{{{\text{Pd}}}}$	_HC Twin_
		Cu layer	400 Å	400 Å	$18 \times \frac{1}{3}[111]a_{{{\text{Cu}}}}$	

The in-plane honeycomb sample is built using the Voronoi construction method with the in-plane single crystal as a representative unit, as shown in Fig. 1b. In HC samples, there are four grains, whose orientation relationships concerning the single crystal (Fig. 1a) are counterclockwise rotation of 25°, 55°, 85° and 0° about the *z*-axis, respectively. The sizes of HC COC and HC Twin are listed in Table 2.

The energy minimization is firstly used to optimize the interfacial structure at 0 K. Then, the relaxation is performed on each sample under the isothermal-isobaric (NPT) ensemble [34, 35] at 300 K for 20 ps to achieve an equilibrium system with zero pressure in *x*-, *y*- and *z*- directions. Uniaxial tension simulations of SC COC and SC Twin along different directions (*x*- or *y*-) with a strain rate of 5 × 10^8^ s^−1^ are performed with the Large-scale Atomic/Molecular Massively Parallel Simulator (LAMMPS) [36]. We also perform tensile simulations of HC COC and HC Twin to study the effects of in-plane GBs and the interfacial structures on their mechanical properties. During loading, the pressures in the other two directions are kept at zero to satisfy the requirement of uniaxial tensile deformation. In all simulations, periodic boundary conditions are applied along the *x-*, *y-* and *z*-directions.

We choose the dislocation extraction algorithm (DXA) [37] to analyze local structures, using which the atoms can be divided into different types (FCC, BCC, HCP, etc.) based on their local structures. It can identify the common dislocations in FCC crystal and determine their Burgers vectors and output dislocation lines [37]. The atoms are colored as the following rule: green for FCC, red for HCP, blue for BCC and white for "other" local crystal structures. It is known that both stacking faults (SFs) and twin boundaries/interfaces (TBs/TIs) are identified as HCP structures, and two adjacent red atomic layers and the single red atomic layer are SF and TB/TI, respectively. An open-source visualization software, OVITO [38], is used to visualize the evolution of microstructures.

## Results and Discussion

### Characterization of interfacial structures

**Fig. 2:**
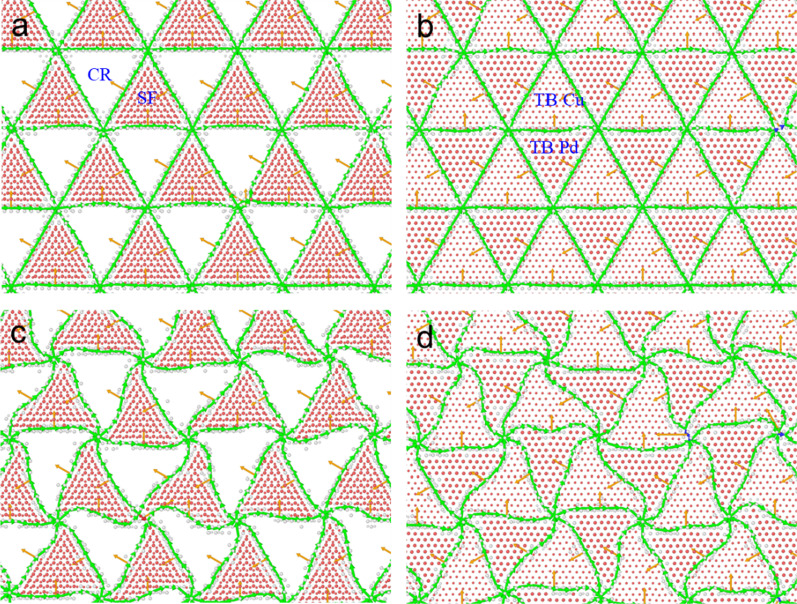
Interfacial atomic configuration after energy minimization: **a** SC COC, **b** SC Twin, and after relaxation: **c** SC COC, **d** SC Twin. The large and small atomic balls represent Pd and Cu, respectively. The atoms identified as FCC have been removed for clarity

Figure 2 shows the interfacial atomic configuration in SC COC and SC Twin after energy minimization and relaxation, where the atoms identified as FCC have been removed for clarity. From Fig. 2, we can see that the interface mismatch dislocation network is triangular in periodicity, which is consistent with that in the Ag(111)/Ni(111) multilayered film [39]. The difference is that the interface in SC COC is composed of alternating coherent regions (CRs) and SF regions. In contrast, the interface in SC Twin is entirely composed of TBs. These TBs are at adjacent atomic layers and are composed of Cu and Pd atoms alternate in adjacent triangles, which can also be confirmed by the height of the two red solid lines (represent the TBs) in the inset of Fig. 1a. During the energy minimization, the potential energy of the system is minimized by the slight movement of atoms, and the size of samples in each direction cannot change freely. In this stage, it is mainly to optimize the local structure, specifically, the interfacial structure. Hence, the dislocation lines remain straight after the energy minimization, as shown in Fig. 2a and b. During the energy minimization, the sample size is fixed, which would induce the residual stresses in all directions. These residual stresses cannot be loosed sufficiently after energy minimization.

During the relaxation, the sample size allows changing to relax the residual stress to zero pressure in all directions. After relaxation, the misfit dislocation lines become bent (Fig. 2c, d). This phenomenon of the misfit dislocation network can also be found in the semi-coherent Cu{111}/Ni{111} interface [40, 41]. By comparing the number of atoms with different local structures, especially HCP, we can find that the number of atoms in different lattice structures changes insignificantly, indicating that the total area of SF and TB varies insignificantly.

To explore whether the temperature is a necessary condition for the bending of dislocation lines, the samples after minimization are relaxed at a low temperature of 10 K for comparison and find that the dislocation lines remain straight. Therefore, a higher temperature is a necessary condition to cause the bending of the dislocation line. Specifically, due to the increased thermal activation at high temperatures, the atoms around the dislocation lines can overturn the energy barrier to move from one atomic column to the adjacent densely packed atomic column. Therefore, the bending magnitude of the dislocation is only one to two atomic layer distances. Similar bending of the dislocation line in the dislocation network can also be observed in the samples with in-plane honeycomb crystals (HC COC and HC Twin).

### Effects of loading direction

**Fig. 3:**
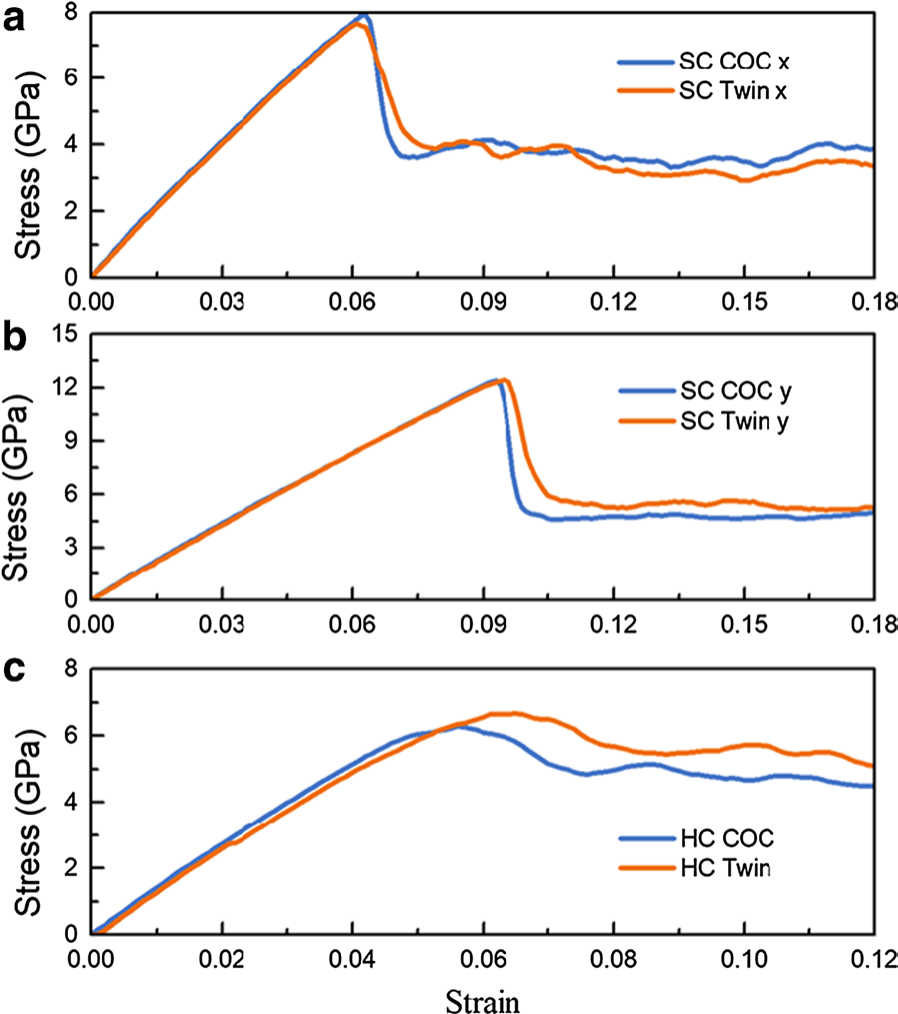
The *σ*-*ε* curves of samples under tension at a strain rate of 5 × 10^8^ s^−1^. SC COC and SC Twin along **a** *x* $[01\overline{1}]$ and **b** *y* $[\overline{2}11]$ direction. **c** HC COC and HC Twin along the *x-*axis

Figure 3 shows the stress–strain (*σ*-*ε*) curves of SC COC and SC Twin under tension along different directions at a strain rate of 5 × 10^8^ s^−1^, where one can see that all these curves grow linearly to the highest point, then drop rapidly to a certain value and fluctuate around them. The young's modulus *E* is obtained by fitting the curves' slope in a strain range of 0.00–0.03, as listed in Table 3*.* We can see that *E* along *y* $[\overline{2}11]$ (145.62 GPa for SC COC and 142.95 for SC Twin) is larger than those along *x* $[01\overline{1}]$ (135.04 GPa for COC and 133.84 GPa for Twin). The *E*s along the same direction but with different interfacial structures are almost identical, showing an insignificant dependence of *E*s on interfacial structures involved in this work, which is consistent with the experimental results of Cu-Co [42], Cu/Pd and Cu/Ni [9] multilayered films.

**Table 3:** *E*, *σ*_m,_ *ε*_m,_ and *σ*_f_ of samples under tension along *x-* or *y*-direction at a strain rate of 5 × 10^8^ s^−1^

	Loading direction	*E* (GPa)	*σ*_m_ (GPa)	*ε*_m_	*σ*_f_ (GPa)
SC COC	*x* $[01\overline{1}]$	135.04	7.93	0.063	3.500.08
SC Twin	*x* $[01\overline{1}]$	133.84	7.66	0.061	3.110.07
SC COC	*y* $[\overline{2}11]$	145.62	12.39	0.093	4.760.07
SC Twin	*y* $[\overline{2}11]$	142.95	12.42	0.095	5.500.12
HC COC	*x*	137.38	6.27	0.056	4.920.16
HC Twin		136.08	6.66	0.065	5.550.08

In a cubic material, the elastic moduli along any orientation can be determined from the elastic constants by application of the following equation [22]:

1 $$\frac{1}{{E_{ijk} }} = S_{11} - 2\left( {S_{11} - S_{12} - \frac{1}{2}S_{44} } \right) \times \left( {l_{i1}^{2} l_{j2}^{2} + l_{j2}^{2} l_{k3}^{2} + l_{i1}^{2} l_{k3}^{2} } \right),$$

where *S*_11_, *S*_12,_ and *S*_44_ are elastic compliance constants; *E*_*ijk*_ is Young’s modulus in the [*ijk*] direction; *l*_i1_, *l*_j2_ and *l*_k3_ are the cosines of the direction [*ijk*]. However, the coefficients about the crystal direction $\left( {l_{i1}^{2} l_{j2}^{2} + l_{j2}^{2} l_{k3}^{2} + l_{i1}^{2} l_{k3}^{2} } \right)$ in Eq. (1) along 〈112〉 and 〈110〉 directions are identical (0.25), therefore, for Cu and Pd, *E*_〈112〉_ = *E*_〈110〉_. When the deformation is parallel to the interface, the mixing rule, $E_{[ijk]}^{{\text{Cu/Pd}}} = E_{[ijk]}^{{{\text{Cu}}}} f_{{{\text{Cu}}}} + E_{[ijk]}^{{{\text{Pd}}}} f_{{{\text{Pd}}}}$, can be used to calculate *E*. *f*_Cu_ and *f*_Pd_ are the volume fraction of Cu and Pd, respectively, and *f*_Cu_ + *f*_Pd_ = 1. In this work, *f*_Cu_ and *f*_Pd_ are invariant for samples with different interfaces. Therefore, $E_{{\left\langle {112} \right\rangle }}^{{\text{Cu/Pd}}}$ should be equal to $E_{{\left\langle {110} \right\rangle }}^{{\text{Cu/Pd}}}$. However, the *E*s along 〈110〉 and 〈112〉 are different, which should be attributed to the elastic anisotropy of the interface-affected zone [6, 42].

The maximum stress (*σ*_m_) obtained by tension along *y*-axis is larger than that along *x*-axis for both COC and Twin interface, which should be ascribed to the Schmidt factor *μ*. The *σ*_m_ of the curve corresponds to the nucleation of dislocation [43–45]. *μ* = cos*φ*cos*λ*, where *φ* and *λ* are the Angle between the tensile direction and the normal direction of the slip plane and the Angle between the tensile direction and the slip direction, respectively. Moreover, when the tension is along *x* $[01\overline{1}]$, the *σ*_m_ and corresponding strain *ε*_m_ of SC COC is slightly higher than that of the SC Twin, which is consistent with the work by Weng et al. [25]. However, when the tension is along *y* $[\overline{2}11]$, the *σ*_m_ and *ε*_m_ of SC COC are slightly lower than that of SC Twin. We further perform additional MD simulations at a lower strain rate of 1 × 10^8^ s^−1^ and obtained similar results. However, overall, the difference between them is slight and can be almost ignored.

After the stress reaches the highest point, many dislocations nucleate successively to release the stored elastic potential energy, causing the rapid drop of stress [46]. The interaction between dislocations, the interaction between dislocations and interface, and the nucleation of new dislocations are the primary mechanism at the flow-stress stage. The *σ*_f_ is the average stress in 0.121 < *ε* < 0.150, as listed in Table 3. Unlike the tiny difference in *E*, *σ*_m_ and *ε*_m_, the difference between the *σ*_f_ for the different interfacial structures is significant. When the tension is along *x* $[01\overline{1}]$, the *σ*_f_ of SC COC is larger than that of SC Twin, showing the strengthening effect of the COC interface is more obvious than that of the Twin interface, which is consistent with the work by Weng et al. [25]. However, when the tension is along *y* $[\overline{2}11]$, the *σ*_f_ of SC Twin is 15.55% larger than that of SC COC, showing an obvious strengthening of the twin interface, which accords with the traditional cognition of strengthening effect of twin boundary. The comparison of flow stress in these two directions shows that the strengthening effect of the interfacial structure depends on the loading direction. In the flowing section, we will examine the mechanical response of in-plane honeycomb crystal samples.

### Effects of in-plane GBs

**Fig. 4:**
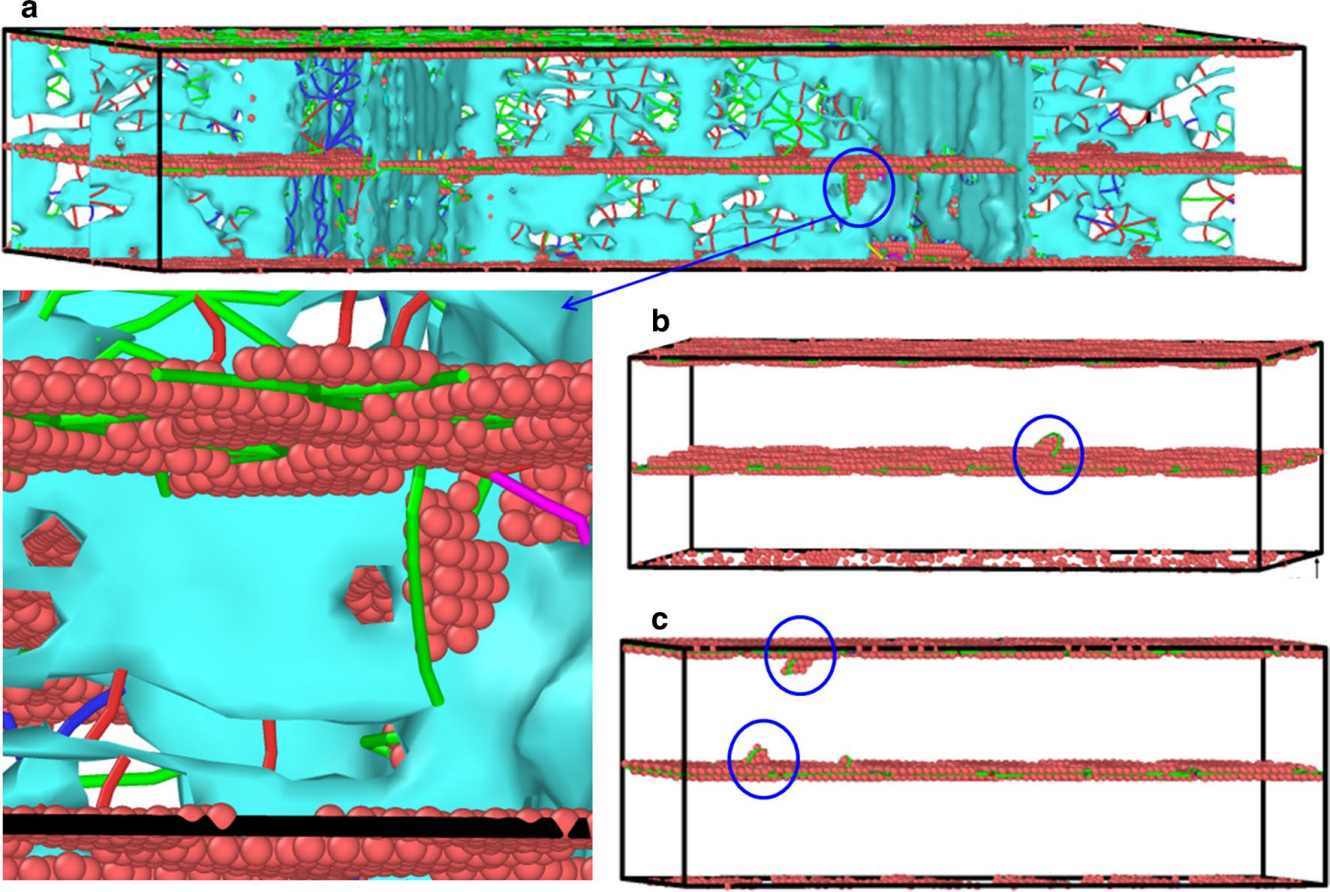
The microstructure of the dislocation nucleation location after the stress reaches the highest point. **a** HC Twin, SC Twin under tension along **b** *x* $[01\overline{1}]$, **c** *y* $[\overline{2}11]$

We further perform MD tension simulation of HC COC and HC Twin at a strain rate of 5 × 10^8^ s^−1^, and the *σ*-*ε* curve is shown in Fig. 3c. Similarly, we can get *E*, *σ*_m_, *ε*_m_, and *σ*_f_, as listed in Table 3. Note that *E* is obtained by fitting the slope of *σ*-*ε* curves of HC COC and HC Twin in a strain range of 0.0–0.02, and *σ*_f_ is the average stress in 0.081 < *ε* < 0.100. For HC COC and HC Twin, the *E*s are close and lie between that of SC sample long the *x* $[01\overline{1}]$ and *y* $[\overline{2}11]$. The *E*s are slightly larger than those by experiment (115–125 GPa)[9], which should be ascribed to the idealized atomic samples used in this work without taking the additional defects such as vacancies and impurities. Their *σ*_m_ is lower than that of the SC sample, which can be ascribed to that the dislocations are easier to nucleate induced by local stress concentration with the introduction of in-plane GBs. Taking the twin interface as an example, Fig. 4 shows the microstructure of the dislocation nucleation location after the stress reaches the highest point, where one can see that in HC Twin, the dislocation nucleates from the junction of the GB and twin interface (Fig. 4a), while in SC Twin samples, the dislocation nucleates from the twin interface both stretched along *x* $[01\overline{1}]$(Fig. 4b) and *y* $[\overline{2}11]$ (Fig. 4c).

Although the *σ*_m_ of the HC sample is lower than those of the SC sample, the *σ*_f_ of the HC sample is higher than the SC sample, indicating the strengthening effect of in-plane GBs. This strengthening mainly comes from the following aspects: (1) The in-plane GBs provides more nucleation points for dislocations resulting in more dislocations nucleated, and these dislocations are hindered by the COC and Twin interface; (2) In-plane GBs hinder dislocations. Moreover, *σ*_f_ of HC Twin is higher than those of HC COC, which shows that the strengthening effects of dislocation hindered by twin interface are more evident than those by COC interface.

**Fig. 5:**
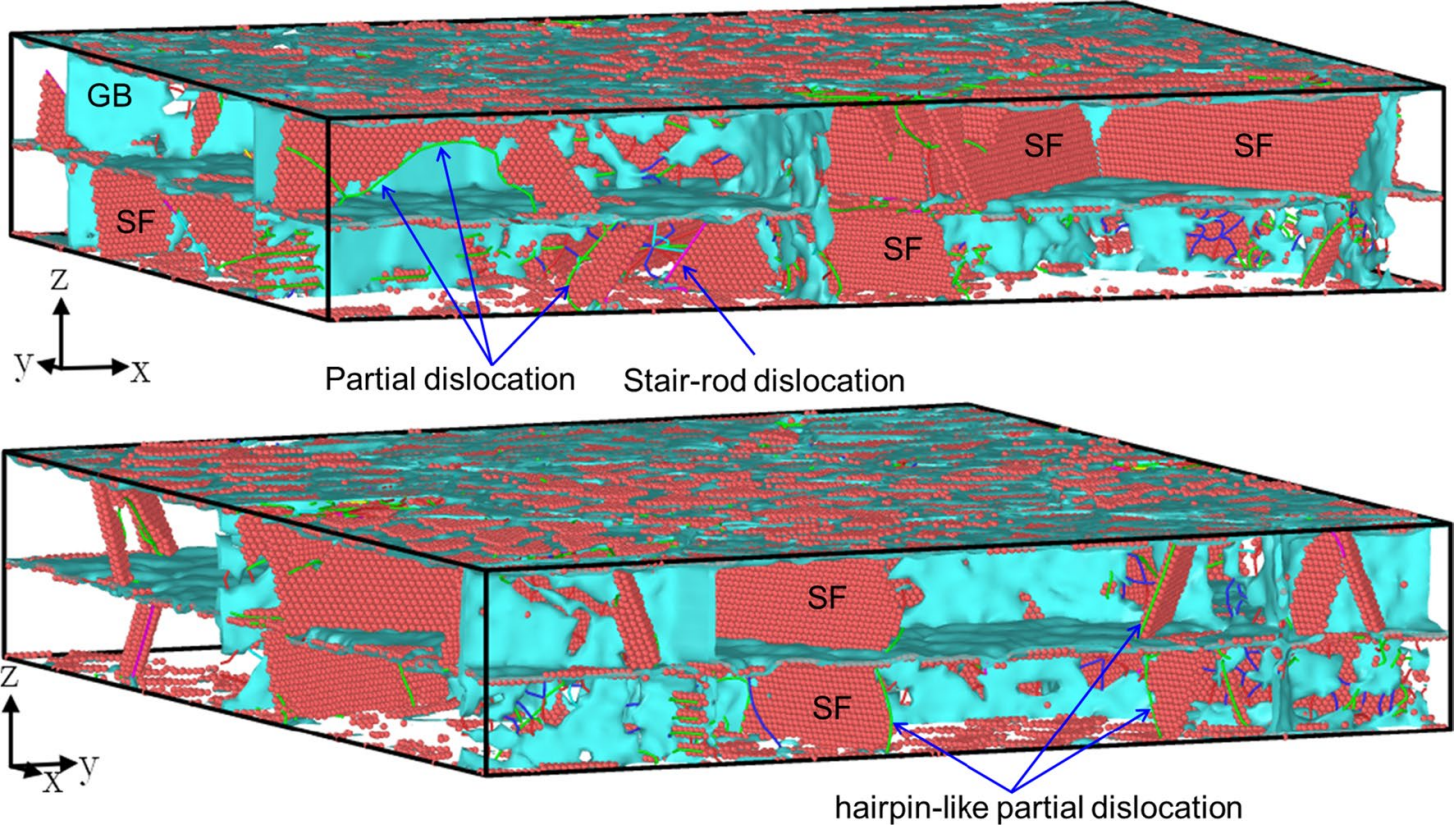
The microstructure of HC Twin at the plastic flow stage

Figure 5 shows the microstructure of HC Twin at the plastic flow stage. It should be noted that during the loading, the nucleation and slip of partial dislocations forming SFs, the movement of these dislocations and SFs limited by the interface inducing hairpin-like partial dislocation glide and the mutual reactions of partial dislocations forming stair-rod dislocation are the primary deformation mechanism. No necklace-like multiple jogged dislocations are observed, which are often observed in Cu/Ni multilayered film [21] and nano-twinned Cu [20] under in-plane tension. It is mainly due to the large lattice mismatch of the Cu/Pd multilayered film with a more complicated interface structure (Fig. 2).

**Fig. 6:**
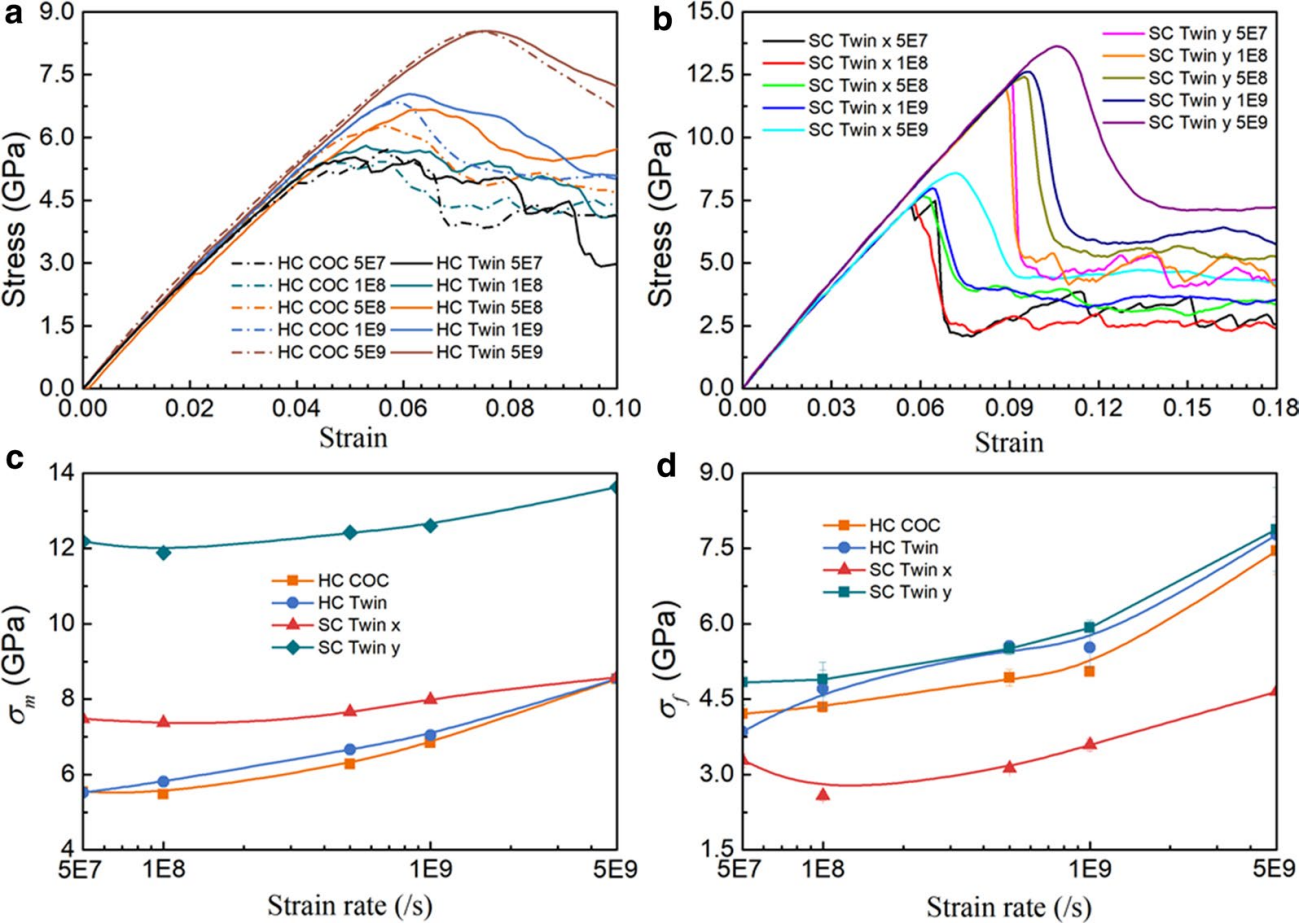
**a** *σ*-*ε* curves of HC samples under tension along *x-*direction at different strain rates, **b** *σ*-*ε* curves of SC Twin under tension along *x-* and *y-*direction at different strain rates. **c-d** Variations of *σ*_m_ and *σ*_f_ against strain rate

Compared with single-crystal materials, the mechanical properties of polycrystalline samples are often more dependent on the strain rate. Therefore, we perform more MD simulations of tension for HC samples (HC COC and HC Twin) along *x*-direction and SC Twin along *x-* and *y*-directions using a strain rate varied from 5 × 10^7^ s^−1^ to 5 × 10^9^ s^−1^. The *σ*-*ε* curves are shown in Fig. 6a and b, where one can see that the stress increases linearly to the highest point and then decreases. For the HC samples, the stress fluctuates with the increase of strain at low strain rate in the descending stage, while the stress fluctuation is not apparent at a high strain rate (Fig. 6a and b). Figure 6c and d shows the variations of *σ*_m_ and *σ*_f_ against strain rate, where *σ*_m_ and *σ*_f_ increase with increasing strain rate. The *σ*_m_ of SC Twin along *y*-direction is much larger than that of other samples, which should be ascribed to the Schmidt factor μ mentioned above. However, due to the strengthening effect of the in-plane grain boundary, the *σ*_f_ of HC samples are closed to that of SC Twin along *y* direction. Moreover, *σ*_f_ of the samples with the twinned interface are higher than those with the COC interface at high strain rate (1 × 10^8^ s^−1^ to 5 × 10^9^ s^−1^), indicating the strengthening effect of the twinned interface, but as the strain rate increases, this strengthening effect weakens. It should be noted that at the strain rate of 5 × 10^7^ s^−1^, the *σ*_f_ of HC Twin is lower than that of HC COC, which may be ascribed to the fact that the number of dislocations nucleated at low strain rate is less inducing the weaken strengthening effect of twin interface.

## Conclusions

In this work, molecular dynamics tension simulations of in-plane single and polycrystalline Cu/Pd multilayered films with COC and twinned interfaces were performed along various directions to explore the effects of the interfacial structure, loading direction and in-plane grain boundaries on the mechanical properties. We found that the interfacial misfit dislocations present a triangular network structure, and the misfit dislocations lines bend after relaxation. The high temperature of 300 K was a necessary condition for the bending of the dislocation line. The elastic modulus of the sample has no obvious dependence on the interface structure, but it is related to the loading direction. The strengthening effect of the COC interface is noticeable when stretched along the 〈110〉 direction; however, the strengthening effect of the twin interface is visible, when stretched along the 〈112〉 direction, showing the anisotropic effect of interfacial structure on mechanical properties. Finally, in the in-plane honeycomb polycrystalline model, the twin interface showed a pronounced strengthening effect, and no jogged dislocations were observed.

## Data Availability

The datasets used or analyzed during the current study are available from the corresponding authors on reasonable request.

## Abbreviations

Cu: Copper
Pd: Palladium
Ni: Nickel
Ag: Silver
COC: Cube-on-cube
NMM: Nanostructured metallic multilayered
GB: Grain boundary
MD: Molecular dynamics
2NN MEAM: Second nearest-neighbor modified embedded atom method
FCC: Face-centered cubic
BCC: Body-centered cubic
HCP: Hexagonal close-packed
SC: Single-crystal
HC: Honeycomb crystal
LAMMPS: Large-scale Atomic/Molecular Massively Parallel Simulator
NPT: Constant number of particles, pressure and temperature
DXA: Dislocation extraction algorithm
SF: Stacking fault
TB: Twin boundary
TI: Twin interface
*σ*–*ε*: Stress–strain
*E*: Young's modulus
*σ*_m_: Maximum stress
